# Rapid Adaptation of Established High-Throughput Molecular Testing Infrastructure for Monkeypox Virus Detection

**DOI:** 10.3201/eid2809.220917

**Published:** 2022-09

**Authors:** Dominik Nörz, Hui Ting Tang, Petra Emmerich, Katja Giersch, Nicole Fischer, Stephan Schmiedel, Marylyn M. Addo, Martin Aepfelbacher, Susanne Pfefferle, Marc Lütgehetmann

**Affiliations:** University Medical Center Hamburg-Eppendorf, Hamburg, Germany (D. Nörz, H.T. Tang, K. Giersch, N. Fischer, S. Schmiedel, M.M. Addo, M. Aepelbacher, S. Pfefferle, M. Lütgehetmann);; Bernhard-Nocht Institute for Tropical Medicine, Hamburg (P. Emmerich, M.M. Addo, S. Pfefferle);; German Center for Infection Research, Hamburg (M.M. Addo)

**Keywords:** monkeypox virus, viruses, zoonoses, SARS-CoV-2, molecular diagnostics, high-throughput PCR testing, cobas, Germany

## Abstract

Beginning in May 2022, a rising number of monkeypox cases were reported in non–monkeypox-endemic countries in the Northern Hemisphere. We adapted 2 published quantitative PCRs for use as a dual-target monkeypox virus test on widely used automated high-throughput PCR systems. We determined analytic performance by serial dilutions of monkeypox virus reference material, which we quantified by digital PCR. We found the lower limit of detection for the combined assays was 4.795 (95% CI 3.6–8.6) copies/mL. We compared clinical performance against a commercial manual orthopoxvirus research use only PCR kit by using clinical remnant swab samples. Our assay showed 100% positive (n = 11) and 100% negative (n = 56) agreement. Timely and scalable PCR tests are crucial for limiting further spread of monkeypox. The assay we provide streamlines high-throughput molecular testing for monkeypox virus on existing broadly established platforms used for SARS-CoV-2 diagnostic testing.

In May 2022, an unusually high number of monkeypox cases were reported in countries in western Europe and North America; by May 29, 2022, 257 laboratory-confirmed infections were reported from Spain, Portugal, the United Kingdom, Canada, and the United States, sparking fear of another global outbreak on the heels of the continuing SARS-CoV-2 pandemic (*1*–*4*). Endemic transmission of the monkeypox virus (MPXV), a species of the *Orthopoxvirus* genus, is thought to be limited to central and western Africa, where both zoonotic (≈22%–72% of cases) and person-to-person transmission contribute to disease burden (*5*). Previous clusters outside Africa have usually been traceable to animal sources rather than to human-to-human transmission (*6*). In contrast, the 2022 cases seem to have occurred without any links to animal sources and have been concentrated in, but not exclusive to, men who have sex with men (*7*). The sudden appearance of infections in several non–monkeypox-endemic countries suggested that undetected transmission might have taken place for some time but that recent events could have served as a catalyst for spread (*1*).

The ongoing SARS-CoV-2 pandemic has demonstrated the potential and value of highly automated high-throughput molecular testing in outbreak scenarios. We aimed to rapidly adapt existing automated molecular testing infrastructure for SARS-CoV-2 in a large tertiary-care hospital in Hamburg, Germany, for detection of MPXV from clinical samples, thereby creating the capacity for high-throughput testing and quick turnaround times, if needed.

## Materials and Methods

### Multiplex Assay Setup 

On the basis of diagnostic testing during the SARS-CoV-2 pandemic (*8*; C. Manohar et al., unpub. data, https://doi.org/10.1101/2021.10.13.21264919), we chose a dual-target approach, in which 1 assay targets a conserved sequence of the *Orthopoxvirus* genus, not including variola major or minor viruses (*9*), and the other targets a MPXV-specific sequence (*10*) (Table 1). The cobas 5800, 6800, and 8800 systems (Roche Diagnostics, https://diagnostics.roche.com) use a spike-in RNA full process control that is added automatically during extraction. The corresponding internal control assay is preloaded in the open channel reagent for use with cobas omni Utility Channel (Roche Diagnostics) (Table 2). We modified and optimized all assays for use on cobas 5800, 6800, and 8800 systems, including 2′O-methyl-RNA-modified primers and internal quenchers for TaqMan probes, as previously described (*11*).

**Table 1:** Primer and probe sequences for a dual-target MPXV assay rapidly adapted from established high-throughput SARS-CoV-2 molecular testing infrastructure*

Oligotype	Oligo name	Sequence, 5′ → 3′	Final concentration, nM
Primers	NVAR fwd	TCA ACT GAA AAG GCC ATC TAT (2'OMe-G)A	400
	NVAR rev	GAG TAT AGA GCA CTA TTT CTA AAT CC(2'OMe-C) A	400
	MPOX fwd	ACG TGT TAA ACA ATG GGT GA(2'OMe-U) G	400
	MPOX rev	AAC ATT TCC ATG AAT CGT AGT (2'OMe-C)C	400
Probes	NVAR P-YAK	YakYellow-CCA TGC AAT (BHQ1)ATA CGT ACA AGA TAG TAG CCA AC-BHQ1	75
	MPOX P-FAM	FAM-TGA ATG AAT (BHQ1)GCG ATA CTG TAT GTG TGG G-BHQ1	75

*Sequences are derived from previously published NVAR assay by Li et al. (*9*) and MPXV assay by Shchelkunov et al. (*10*) and adapted for the cobas omni Utility Channel (Roche Diagnostics, https://diagnostics.roche.com). Oligos were custom made by Ella Biotech GmbH (https://www.ellabiotech.com). Indicated final concentration refers to the final oligo concentrations within the reaction mix. 2′O-methyl-RNA bases are indicated as OMe-X. Internal abasic quenchers are indicated as (BHQ1). NVAR, nonvariola orthopoxvirus; MPXV, monkeypox virus.

**Table 2:** Software settings for run protocol for a dual-target MPXV assay rapidly adapted from established high-throughput molecular testing infrastructure*

Protocol setting	Channel setting, use				
	1, Not used	2, Monkeypox	3, Nonvariola	4, Not used	5, Internal control
Relative fluorescence increase	NA	2	2	NA	2
PCR cycling conditions	UNG incubation	Pre-PCR step	1st measurement	2nd measurement	Cooling
No. cycles	Predefined	1	5	45	Predefined
No. steps	Predefined	3	2	2	Predefined
Temperature	Predefined	55°C; 60°C; 65°C	95°C; 55°C	91°C; 58°C	Predefined
Hold time	Predefined	120 s; 360 s; 240 s	5 s; 30 s	5 s; 25 s	Predefined
Data acquisition	Predefined	None	End of each cycle	End of each cycle	Predefined


*Protocol was run on 400 µL swab samples. Protocol adapted for cobas omni Utility Channel (Roche Diagnostics, https://diagnostics.roche.com). Relative fluorescence increase thresholds were used for automated result calls. NA, not applicable; UNG, uracyl-N-glycosylase.

### In Silico Evaluation

As part of a support request for Utility Channel applications, we submitted all sequences of the duplex assay to Roche Diagnostics for evaluation of inclusivity and potential primer-probe interactions. The submitted sequences were aligned to currently available MPXV and orthopoxvirus sequences available in public databases.

### Analytical Performance Evaluation

We conducted technical performance evaluations for the assays according to new European Union regulations (Regulation 2017/746 EU IVDR, https://euivdr.com). For reference material, we used inactivated cell culture supernatant containing MPXV recovered from a clinical case in central Africa in 1987 (*12*) and inactivated modified vaccinia virus Ankara (MVA) SARS-CoV-2 vaccine. To obtain a quantitative MPXV standard, we purified nucleic acids by using a MagNA-pure96 extractor and Viral NA Small Volume Kit (Roche Diagnostics) and analyzed on a QIAcuity digital PCR instrument (QIAGEN, https://www.qiagen.com) in conjunction with 3 different previously described quantitative PCRs (qPCRs): 1 for nonvariola (NVAR) orthopoxviruses (*9*); 1 for MPXV (*10*); and the research-use only (RUO) LightMix Modular Orthopoxvirus Assay (TIB MOLBIOL, https://www.tib-molbiol.de).

We determined the lower limit of detection by serial 2-fold dilution of MPXV standard in universal transport medium from 100 to 0.78 copies/mL and 21 repeats per dilution step. Using MedCalc statistical software (https://www.medcalc.org), we determined the limit for 95% probability of detection. We assessed linearity by 10-fold serial dilution of MPXV standard (5 repeats per dilution step) at concentrations of ≈10^1^–10^7^ copies/mL. We determined linearity and intra-assay variability by using Validation Manager software (Finbiosoft, https://finbiosoft.com). Concentrations represent copies per mL of specimen. For empirical inclusivity and exclusivity testing, we used the assay to test a set of 53 samples, including clinical samples, reference material, and external quality controls from a range of bloodborne and respiratory pathogens (Appendix Table 1). We used an experimental MVA vector-based SARS-CoV-2 vaccine as reference material for a non-MPXV orthopoxvirus.

### Clinical Evaluation and Follow-Up Samples

For clinical validation, we used the RUO LightMix Modular Orthopoxvirus assay as reference test, which we performed according to manufacturer’s recommendation by using the MagNA-pure96 system with 200-µL extraction volume. In total, we tested 67 clinical samples consisting of respiratory, skin, and genital swab samples with both assays. Of those samples, 11 were positive for MPXV DNA, which we obtained from 2 confirmed clinical cases in Hamburg, Germany. We analyzed 33 consecutive clinical samples from the same 2 patients and 2 additional cases by using the duplex assay (Appendix Table 2). The clinic provided globalized patient characteristics.

## Results

### In Silico Analysis

We did not detect any concerning oligo interactions (Appendix Figure 1). Target-1: NVAR was still a 100% match for all but 1 MPXV sequence, which had 1 low-risk mismatch. NVAR also had high sequence similarity with many other orthopoxviruses but might not be optimal for reliable detection of camelpox or cowpox (Appendix Figure 2). Target-2: MPOX is a perfect match for almost all Congo Basin strain MPXV sequences but has a known mismatch for West Africa strain sequences in the probe region. This mismatch is expected to slightly reduce relative fluorescence increase signals, as demonstrated in the clinical sample set. Other orthopoxviruses have extensive sequence mismatches with this assay and are not expected to produce detectable signals (Appendix Figure 3).

### Analytical performance

**Figure 1:**
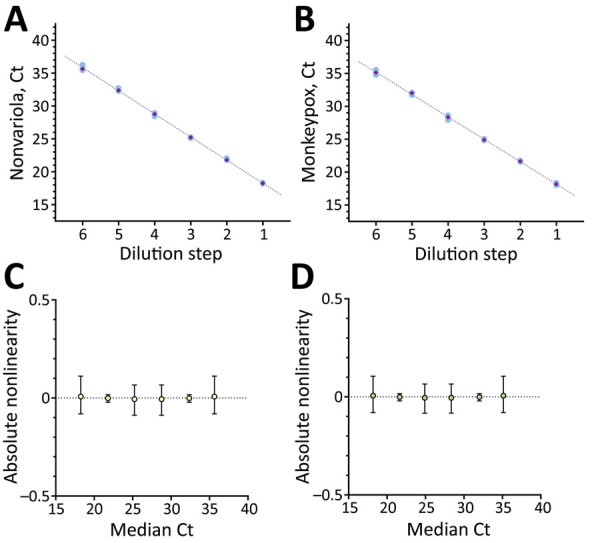
Linearity data for the dual-target monkeypox virus assay rapidly adapted from established high-throughput molecular testing infrastructure. A) Nonvariola orthopoxvirus target; B) monkeypox virus target; C) absolute Ct for nonvariola orthopox virus target; D) absolute Ct for monkeypox virus target. Linearity was determined by serial dilution of monkeypox virus reference material from cell culture supernatant of Congo Basin monkeypox strain collected in 1987. Analysis was performed on Validation Manager software (Finbiosoft, https://finbiosoft.com). Nonvariola orthopoxvirus slope was −3.52, r^2^ = 0.999; monkeypox virus slope was −3.40, r^2^ = 0.999. Ct, cycle threshold.

We determined LoD was 9.697 (95% CI 7.424–15.327) copies/mL for the NVAR assay and 6.359 (95% CI 4.908–10.110) copies/mL for the MPOX assay by probability of detection analysis. Overall LoD for both targets combined was 4.795 (95% CI 3.598–8.633) copies/mL. We compiled hit rates (Table 3) and probability of detection plots (Appendix Figure 4) for the assay. The assay showed excellent linearity. Cycle threshold (Ct) values were 37–18, ≈10^1^–10^7^ copies/mL, and pooled SD and 95% CI were within linear range: Ct 0.194, SD 0.0662% for NVAR; Ct 0.175, SD 0.618% for MPXV (Figure 1).

**Table 3:** Hit rates during limit of detection studies of dual-target MPXV assay rapidly adapted from established high-throughput molecular testing infrastructure*

Concentration, copies/mL	NVAR	MPXV	Overall
100	21/21	21/21	21/21
50	21/21	21/21	21/21
25	21/21	21/21	21/21
12.5	20/21	21/21	21/21
6.25	19/21	20/21	21/21
3.125	11/21	13/21	15/21
1.56	11/21	11/21	17/21
0.78	3/21	6/21	7/21

*Results represent no. positive/no. tested. Limits of detection were determined by serial dilution of a quantified MPXV standard (quantified by digital PCR) as a reference. Concentrations represent copies/mL of specimen. Dilution series were generated automatically using a STARlet Liquid Handler (Hamilton, https://www.hamiltoncompany.com). We calculated 95% probability of detection by using MedCalc statistical software (https://www.medcalc.org). MPXV, monkeypox virus; NVAR, nonvariola orthopoxvirus.

No false positives occurred within the inclusivity-exclusivity set. The MVA vector vaccine was correctly detected by the NVAR assay, and not by the MPXV assay (Appendix Table 1).

### Clinical Evaluation

**Figure 2:**
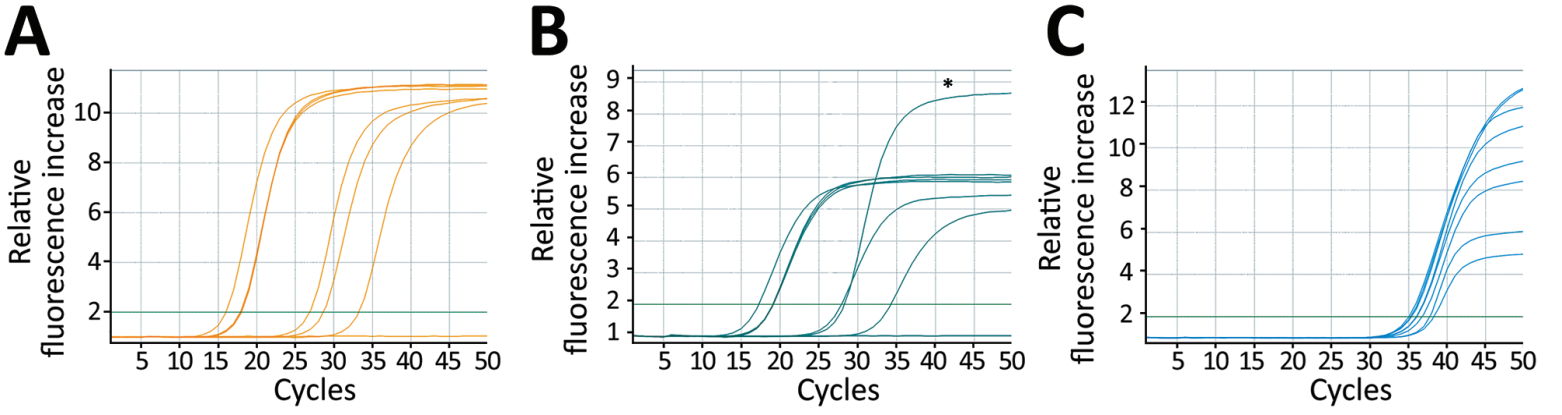
Amplification curves of clinical samples, including internal controls for dual-target monkeypox virus assay rapidly adapted from established high-throughput molecular testing infrastructure. A) Nonvariola orthopoxvirus; B) monkeypox virus; C) internal control. Samples included clinical swab specimens of monkeypox lesions, oropharyngeal swab samples, and EDTA plasma from patients with confirmed monkeypox, Hamburg, Germany. Asterisk (*) in panel B indicates the positive control curve in channel 2, which was the cell culture supernatant of Congo Basin monkeypox strain collected in 1987. West Africa strain samples exhibit a reduction of approximately one third in relative fluorescence increase for monkeypox virus, due to a known mismatch in the probe region (Appendix Figure 1).

In total, we tested 67 clinical samples, consisting of respiratory, skin, and genital swab samples, with both assays. Of those, 11 samples obtained from 2 confirmed clinical monkeypox case-patients in Hamburg, Germany, were positive for MPXV DNA. We noted 100% positive (11/11) and 100% negative agreement (56/56) for the 2 assays (Figure 2).

### Results from Different Sample Types and Timepoints

Another 33 clinical samples were longitudinally collected from 4 patients, all of whom were male, 20–40 years of age, and had 6–50 skin lesions; 1 patient had known HIV infection under treatment. Lesion swabs generated Ct values of 13.3–16.1, oropharyngeal swabs Ct values of 13.1–33.3, and blood samples Ct values of 30.3–38.4. A small sample set of urine had only low concentrations of viral DNA, Ct 31.1–37.8. A single patient provided seminal fluid, which had Ct values of 32.9 for the NVAR assay and 33.9 for the MPXV assay when diluted in guanidine hydrochloride solution (Appendix Table 2).

## Discussion

The trajectory of the ongoing MPXV outbreak in Europe and North America has many uncertainties. However, the World Health Organization acknowledges that known clusters represent a change in transmission pattern and emphasizes the need to limit further spread (*1*). Broad availability of molecular testing with short turnaround times is a crucial prerequisite for reducing monkeypox spread.

We adapted 2 established nonvariola orthopoxvirus and MPXV qPCR assays (*9*,*10*) as a duplex test for the cobas 5800, 6800, and 8800 fully automated sample-to-result platforms, which are widely used for high-throughput SARS-CoV-2 diagnostic testing (*13*). Both assays have been validated extensively against other orthopoxvirus species in previous studies (*9*,*10*) and remain inclusive and highly specific for in silico analysis with currently available monkeypox sequences. We demonstrated excellent analytical performance of the duplex assay, showing single-digit detection limits and near-perfect PCR efficiency. A spike-in full-process control assay, similar to commercial in vitro diagnostic assays, already is included in the open channel reagents we used.

Our institution confirmed 4 clinical cases of monkeypox, and we used the initial clinical samples as our clinical positive set. Although the assay was only validated on swab samples, we also detected MPXV DNA in EDTA plasma, urine, and seminal fluid diluted in guanidine hydrochloride solution without any method adaptations. Among all tested clinical samples positive for MPXV DNA, swabs of skin lesions consistently yielded early Ct values in the low- to mid-teens, indicating exceedingly high viral DNA loads, which might be a concern for both personnel safety and contamination risks. Viral DNA was readily detectable in oropharyngeal swab samples, as previously reported (*2*,*14*). Likewise, EDTA plasma samples were consistently positive but had later Ct values, mostly around 30. Further studies could evaluate the practical usefulness of plasma or urine for monkeypox diagnostic purposes or longitudinal viral load monitoring. Our data regarding MPXV DNA in different clinical specimen types are well in line with other published studies (*2*,*14*); overall, skin lesion swab samples appeared to be best suited for diagnostic purposes based on our sample set.

In conclusion, we provided technical performance evaluation for a laboratory-developed duplex qPCR assay for MPXV detection for use on the cobas 5800, 6800, and 8800 high-throughput systems. The assay we describe enables laboratories to adapt existing automated SARS-CoV-2 molecular testing infrastructure for a potential large-scale monkeypox outbreak.

Appendix Additional information on rapid adaptation of established high-throughput SARS-CoV-2 molecular testing infrastructure for monkeypox virus detection.
