# Supplementary material for: Rapid Adaptation of Established High-Throughput Molecular Testing Infrastructure for Monkeypox Virus Detection
Source: Emerg Infect Dis. 2022 Sep;28(9):1765–9. doi: 10.3201/eid2809.220917 (PMC9423910; doi:10.3201/eid2809.220917)
Supplement: Appendix — Additional information on rapid adaptation of established high-throughput SARS-CoV-2 molecular testing infrastructure for monkeypox virus detection. [file 22-0917-Techapp-s1.pdf]

Article DOI: https://doi.org/10.3201/eid2809.220917

Rapid Adaptation of Established High-Throughput Molecular Testing Infrastructure for Monkeypox Virus Detection

Appendix

Appendix Table 1. Clinical samples, reference material, and external control panels used for the inclusivity and exclusivity set for rapid adaptation of established high-throughput molecular testing infrastructure for monkeypox virus detection*

		Target	
Species	No. tested	Nonvariola orthopoxvirus	Monkeypox virus
External reference material, quality control panel			
Monkeypox virus (Congo Basin, cell culture, 1987)	3	**Positive**	**Positive**
Vaccinia virus Ankara strain SARS-2-ST_HD	1	**Positive**	Negative
Influenza-B (INSTAND e.V.)	1	Negative	Negative
Parainfluenzavirus-1 (Zeptometrix)	1	Negative	Negative
Parainfluenzavirus-4 (Zeptometrix)	1	Negative	Negative
Human coronavirus OC43 (Zeptometrix)	1	Negative	Negative
Clinical samples			
Herpes simplex virus 1	5	Negative	Negative
Varicella-zoster virus	4	Negative	Negative
Cytomegalovirus	5	Negative	Negative
Ebola virus	5	Negative	Negative
Human herpesvirus 6	5	Negative	Negative
BK virus	5	Negative	Negative
John Cunningham virus	4	Negative	Negative
SARS-CoV-2	1	Negative	Negative
Influenza A	1	Negative	Negative
Respiratory syncytial virus	1	Negative	Negative
Parainfluenzavirus-3	1	Negative	Negative
Rhinovirus/enterovirus	3	Negative	Negative
Adenovirus	2	Negative	Negative
Bocavirus	2	Negative	Negative
Human coronavirus HKU1	1	Negative	Negative

*No false positives occurred. The MVA-SARS-2 vacciniavirus–based vaccine (Ankara strain) was correctly detected by the nonvariola orthopoxvirus assay and not by the monkeypox virus assay.

Appendix Table 2. Clinical samples tested with the dual-target monkeypox virus assay rapidly adapted from established high-throughput SARS-CoV-2 molecular testing infrastructure*

		Target, cycle threshold	
Sample type	Admission day	Nonvariola orthopoxvirus	Monkeypox virus
Lesion swab	1	15	15.5
Lesion swab	1	14.9	15.8
Lesion swab	1	13.3	14.7
Lesion swab	2	14.3	13.9
Lesion swab	2	13.5	14.7
Lesion swab	3	13.9	15.3
Lesion swab	4	15.3	15.7
Lesion swab	5	14.3	15.4
Lesion swab	6	15.2	16.1
Lesion swab	7	14.4	15.6
Lesion swab	9	13.9	15.2
Oropharyngeal swab	1	23.8	24.2
Oropharyngeal swab	1	13.1	13.8
Oropharyngeal swab	1	17.1	18.4
Oropharyngeal swab	2	24.1	24.6
Oropharyngeal swab	2	21.9	23
Oropharyngeal swab	3	22.4	23.6

Sample type	Admission day	Target, cycle threshold	
		Nonvariola orthopoxvirus	Monkeypox virus
Oropharyngeal swab	4	20.5	20.9
Oropharyngeal swab	5	26.9	28.08
Oropharyngeal swab	6	29.3	30.6
Oropharyngeal swab	7	27.9	28.9
Oropharyngeal swab	9	32.1	33.3
EDTA plasma	1	30.5	30.4
EDTA plasma	2	31.3	31.4
EDTA plasma	2	30.3	31.1
EDTA plasma	3	33.4	33.6
EDTA plasma	4	38.4	Not detected
EDTA plasma	4	30.7	31.5
EDTA plasma	6	36.1	37.2
EDTA plasma	8	Not detected	38
Urine	2	37.6	37.8
Urine	5	31.32	32.58
Seminal fluid	4	32.9	33.9

*Follow-up patient samples were analyzed with the dual-target assay. Specimens were collected from 4 different patients with previously confirmed monkeypox virus infection in Hamburg, Germany.

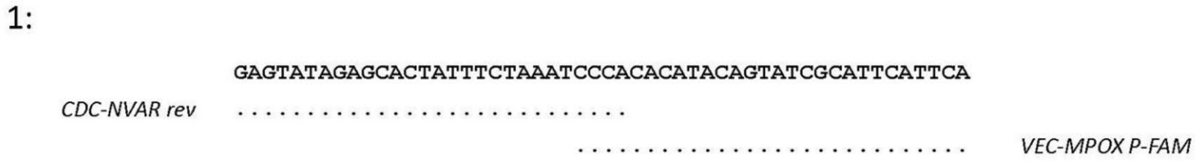

Appendix Figure 1. Potential oligo-oligo interaction of the dual-target monkeypox virus assay rapidly adapted from established high-throughput molecular testing infrastructure. The NVAR reverse primer (*1*) and MPOX probe (*2*) show a 3′ overlap of 4 bases. However, the interaction cannot create dimers on its own. Similar interactions can, in rare cases, create a viable dimer in a secondary unspecific amplification event, thereby also leading to unspecific signals. The risk for such an event is further mitigated by 2′Omethyl-RNA modified primers and internal quenchers. No unspecific signals were observed in wetlab experiments. Unspecific signals of the MPOX assay would yield nonsensical results (i.e., NVAR negative, MPOX positive), prompting further investigation. Based on these factors, the interaction was deemed not significant. MPOX, monkeypox virus; NVAR, nonvariola orthopox virus.

Target-1: NVAR

group	n(Sequences)	hits	forward-1(f)<br>TCAACTGAAAAGGCCATCTATGA	probe-1(p)<br>CCATGCAATATACGTACAAGATAGTAGCCAAC	reverse-1(r)<br>GAGTATAGAGCACTATTTCTAAATCCCA
Monkeypox	**n = 105**	1	....	....	....
Monkeypox	n = 1	1	.................T.....	....	....
Abatino_macacapox	n = 2	1	....	....	..............G.............
Akhmeta	n = 5	1	..C....................	....	..............G.............
Akhmeta	n = 2	1	..C........A...........	.............A.....A..C.........	....
Camelpox	n = 10	1	....	.......G..............C.........	....
Cowpox	n = 29	1	....	....	....
Cowpox	n = 2	1	....	......................C.........	........C..G................
Cowpox	n = 5	1	....	....	........C..G................
Cowpox	n = 1	1	....	.......G..............C.........	...........G................
Cowpox	n = 1	1	....	.......G..............C..G......	....
Cowpox	n = 5	1	....	....	..............G.............
Cowpox	n = 1	1	....	......................C.........	..............G.............
Cowpox	n = 2	1	..............T........	.......G..............C.........	....
Cowpox	n = 5	1	....	....	........C..G................
Ectromelia	n = 13	1	....	....	..............G.............
Raccoonpox	n = 5	1	..T....................	.............A.....A.....T..G..T	.....G......................
Skunkpox	n = 4	1	..T........A...........	.............T..T..A........G..T	.....G......................
Taterapox	n = 3	1	....	.......G..............C.........	....
Vaccinia	n = 31	1	.................T.....	....	....
Vaccinia	n = 1	1	......A................	..........G................A....	A...........................
Vaccinia	n = 72	1	....	....	....
Vaccinia	n = 11	1	.................T.....	...................A............	....
Vaccinia	n = 11	1	....	...................A............	....
Variola	n = 57	1	....	.......G..............CA........	....
Volepox	n = 4	1	..T........A.....A.....	.A...........T..T..A........G..T	.....G......................

Appendix Figure 2. Primer and probe alignments for the NVAR target of the dual-target assay rapidly adapted from established high-throughput SARS-CoV-2 molecular testing infrastructure. Alignments were provided by Roche Diagnostics (https://diagnostics.roche.com) as part of a support request. Currently available orthopoxvirus sequences were checked for mismatches against the NVAR assay. The number of sequences represented by each line is indicated on the left. A single monkeypox virus sequence with 1 primer mismatch is noted, with a low risk for relevant effect. NVAR, nonvariola orthopoxvirus

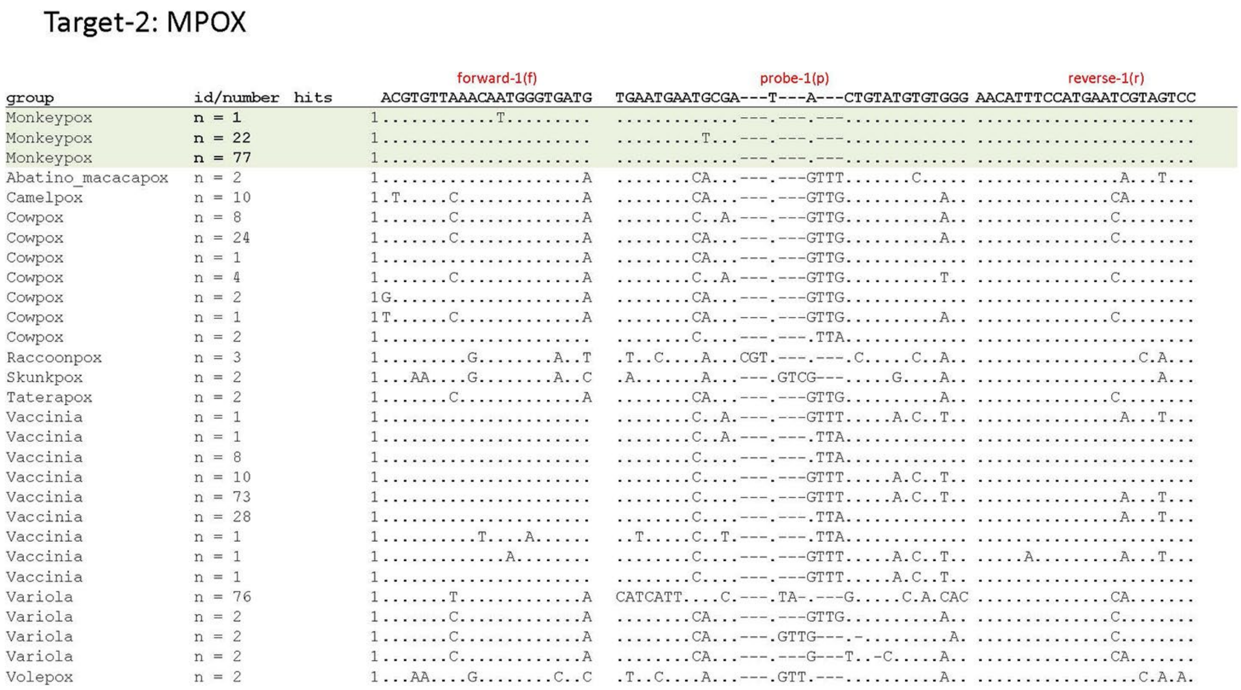

Appendix Figure 3. Primer and probe alignments for the MPOX of the dual-target monkeypox virus assay rapidly adapted from established high-throughput SARS-CoV-2 molecular testing infrastructure. MPOX alignments were provided by Roche Diagnostics (https://diagnostics.roche.com) as part of a support request. Currently available orthopoxvirus sequences were checked for mismatches against the MPOX assay. The number of sequences represented by each line is indicated on the left. Monkeypox virus of the West Africa strain have a known mismatch in the probe region (G to T), leading to a reduction in RFI of ≈1/3 (Figure 2). MPOX, monkeypox virus; RFI, relative fluorescence increase.

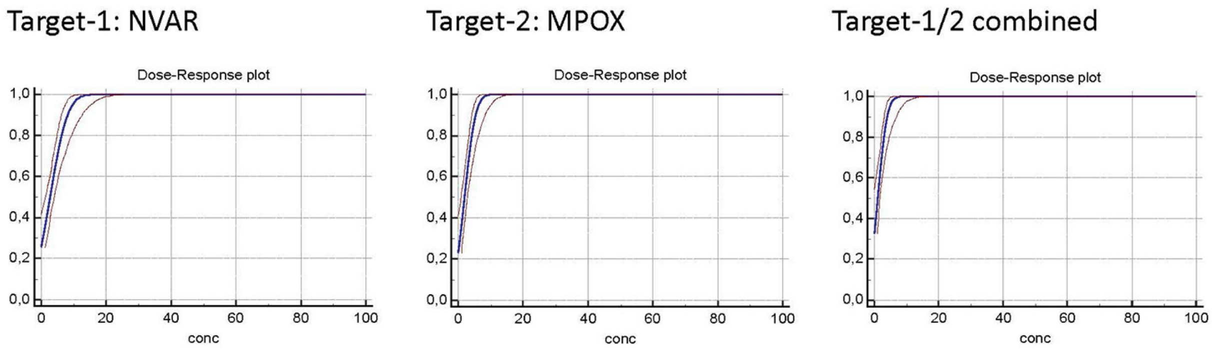

Appendix Figure 4. Probit curves of the lower limit of detection experiment for dual-target monkeypox virus assay rapidly adapted from established high-throughput SARS-CoV-2 molecular testing infrastructure. In brief, a 2-fold dilution series of quantified monkeypox virus standard (quantified by digital PCR) was used to determine the 95% probability of detection (21 repeats per dilution step). Confidence intervals are indicated in red. Hit-rates are displayed in Table 3 in the main text. Conc, concentration; MPOX, monkeypox virus; NVAR, nonvariola orthopoxvirus.

References

1. Li Y, Olson VA, Laue T, Laker MT, Damon IK. Detection of monkeypox virus with real-time PCR assays. J Clin Virol. 2006;36:194–203. PubMed https://doi.org/10.1016/j.jcv.2006.03.012

2. Shchelkunov SN, Shcherbakov DN, Maksyutov RA, Gavrilova EV. Species-specific identification of variola, monkeypox, cowpox, and vaccinia viruses by multiplex real-time PCR assay. J Virol Methods. 2011;175:163–9. PubMed https://doi.org/10.1016/j.jviromet.2011.05.002
